# SARS-CoV-2 D614G variant exhibits efficient replication ex vivo and transmission in vivo

**DOI:** 10.1126/science.abe8499

**Published:** 2020-11-12

**Authors:** Yixuan J. Hou, Shiho Chiba, Peter Halfmann, Camille Ehre, Makoto Kuroda, Kenneth H. Dinnon, Sarah R. Leist, Alexandra Schäfer, Noriko Nakajima, Kenta Takahashi, Rhianna E. Lee, Teresa M. Mascenik, Rachel Graham, Caitlin E. Edwards, Longping V. Tse, Kenichi Okuda, Alena J. Markmann, Luther Bartelt, Aravinda de Silva, David M. Margolis, Richard C. Boucher, Scott H. Randell, Tadaki Suzuki, Lisa E. Gralinski, Yoshihiro Kawaoka, Ralph S. Baric

**Affiliations:** 1 Department of Epidemiology, University of North Carolina at Chapel Hill, Chapel Hill, NC, USA.; 2 Influenza Research Institute, Department of Pathobiological Sciences, School of Veterinary Medicine, University of Wisconsin, Madison, WI, USA.; 3 Marsico Lung Institute, University of North Carolina at Chapel Hill, Chapel Hill, NC, USA.; 4 Department of Microbiology and Immunology, University of North Carolina at Chapel Hill, Chapel Hill, NC, USA.; 5 Department of Pathology, National Institute of Infectious Diseases, Tokyo, Japan.; 6 Department of Medicine, University of North Carolina at Chapel Hill, Chapel Hill, NC, USA.; 7 Division of Virology, Department of Microbiology and Immunology, Institute of Medical Science, University of Tokyo, Tokyo, Japan.

## Abstract

The spike aspartic acid–614 to glycine (D614G) substitution is prevalent in global severe acute respiratory syndrome coronavirus 2 (SARS-CoV-2) strains, but its effects on viral pathogenesis and transmissibility remain unclear. We engineered a SARS-CoV-2 variant containing this substitution. The variant exhibits more efficient infection, replication, and competitive fitness in primary human airway epithelial cells but maintains similar morphology and in vitro neutralization properties, compared with the ancestral wild-type virus. Infection of human angiotensin-converting enzyme 2 (ACE2) transgenic mice and Syrian hamsters with both viruses resulted in similar viral titers in respiratory tissues and pulmonary disease. However, the D614G variant transmits significantly faster and displayed increased competitive fitness than the wild-type virus in hamsters. These data show that the D614G substitution enhances SARS-CoV-2 infectivity, competitive fitness, and transmission in primary human cells and animal models.

## Changing with the times

Pandemic spread of a virus in naïe populations can select for mutations that alter pathogenesis, virulence, and/or transmissibility. The ancestral form of severe acute respiratory syndrome coronavirus 2 (SARS-CoV-2) that emerged from China has now been largely replaced by strains containing the mutation D614G (Asp^614^-to-Gly) in the viral spike protein. Hou *et al.* compared the characteristics of the new variant against the ancestral form in a series of experiments in human cells and animal models. The variant is better at infecting upper-airway epithelial cells and replicates in greater numbers than the ancestral virus. Evidence indicates modest, if any, significant changes to virulence in animal models. Therefore, the virus appears to have evolved for greater transmissibility in humans rather than for greater pathogenicity. The mutation renders the new virus variant more susceptible to neutralizing antisera without altering the efficacy of vaccine candidates currently under development.

*Science*, this issue p. 1464

## Teaser

The current dominant structural variant of SARS-CoV-2 appears to have evolved from the ancestral form and enhances transmissibility.

The expanding coronavirus disease 2019 (COVID-19) pandemic, caused by severe acute respiratory syndrome coronavirus 2 (SARS-CoV-2), has had an unprecedented impact on modern human civilization, resulting in more than 1.1 million deaths globally. Pandemic spread of the virus in naïve populations may select for mutations that alter pathogenesis, virulence, and/or transmissibility. Despite the presence of a CoV proofreading function in viral replication (*1*, *2*), recent reports identified an emergent Asp^614^→Gly (D614G) substitution in the spike glycoprotein of SARS-CoV-2 strains that is now the most prevalent form globally. Patients infected with the D614G variant are associated with higher viral loads in the upper respiratory tract than seen with the ancestral strain, but not with altered disease severity (*3*, *4*). SARS-CoV-2 S pseudotyped viruses encoding the D614G substitution were reported to exhibit increased infectivity in continuous cell lines and increased sensitivity to neutralization (*4*, *5*). Structural analyses also revealed that the receptor binding domains (RBDs) in the G614-form S protein occupy a higher percentage in the open conformation than the D614 form, implying an improved ability to bind to the receptor angiotensin-converting enzyme 2 (ACE2) (*6*, *7*). However, the D614G substitution has yet to be evaluated in the authentic SARS-CoV-2 infection models, and its functions in viral replication, pathogenesis, and transmissibility remain unclear.

**Fig. 1:**
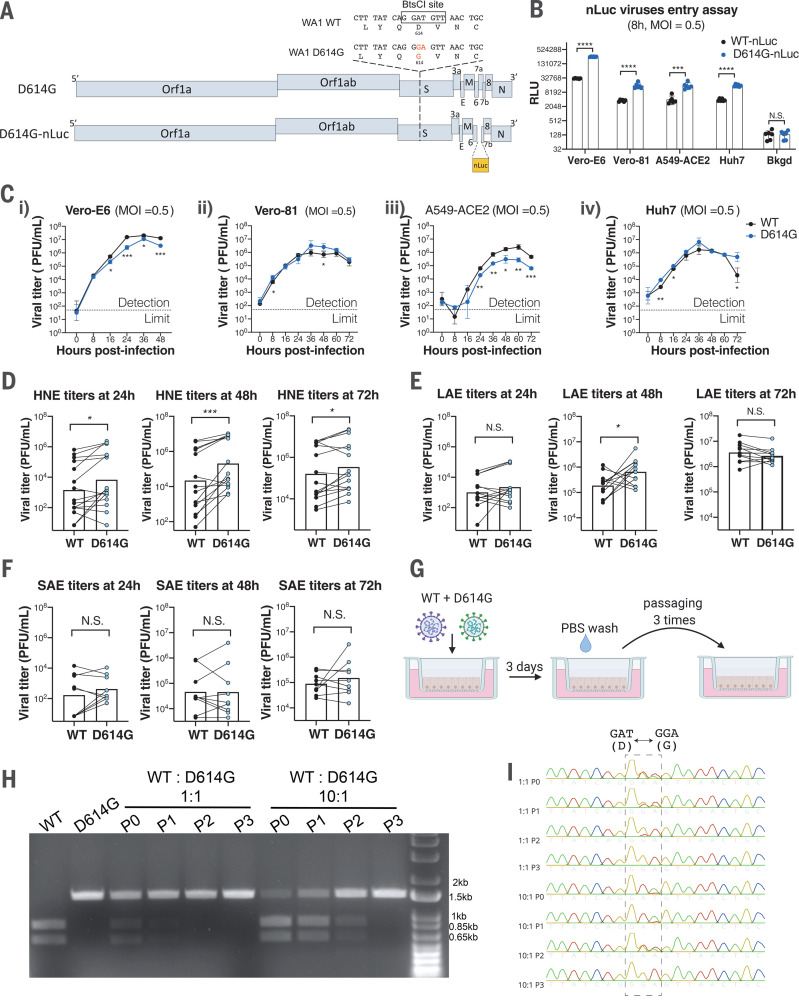
SARS-CoV-2 D614G variant shows enhanced infectivity in immortalized cell lines and replication fitness in upper human respiratory epithelia compared with the ancestral WT virus. (**A**) Genomes of recombinant SARS-CoV-2 D614G variants based on the backbone of a D614-form strain WA1. E, envelope; M, membrane; N, nucleocapsid; nLuc, nanoluciferase; S, spike. (**B**) Entry efficiency of WT-nLuc and D614G-nLuc in multiple susceptible cell lines at a MOI of 0.5. After 1-hour of infection, cells were treated with neutralization Abs to minimize the secondary round of infection. The relative light unit (RLU) representing the nLuc expression level was measured at 8 hours after infection. RLU values were normalized with background (Bkgd) residual luciferase signals in both viral inocula. (**C**) Growth curves of the two viruses in Vero-E6 (i), Vero-81 (ii), A549-ACE2 (iii), and Huh7 (iv) cell lines at a MOI = 0.5. (**D** to **F**) Comparison of 24-, 48-, and 72-hour titers between the two variants in infected primary nasal (D), large airway (E), and small airway (F) cells at a MOI of 0.1. Triplicated titers of the two viruses in the cultures from the same donor were analyzed by paired *t* test. (**G**) Schematic of competition assays on LAE cells. Cultures were infected with a 1:1 or 10:1 ratio of WT and D614G mixture at a MOI of 0.5, and the supernatants were serially passaged three times in naïve cultures. PBS, phosphate-buffered saline. (**H** and **I**) BtsCI digestion (H) and Sanger sequencing chromatogram (I) of S gene fragments amplified from viral samples in the LAE competition assay. The 1.5-kb fragments containing the residue 614 were amplified from the total RNA of individual samples collected in each passage. P, passage. Data in (B) and (C) are indicated as mean ± SD and were analyzed by unpaired *t* test between both viruses; data in (D) to (F) were analyzed by paired *t* test. N.S., not significantly different; *P < 0.05; **P < 0.01; ***P < 0.001; ****P < 0.0001.

To address these questions, we generated an isogenic SARS-CoV-2 variant containing only the D614G substitution in the S glycoprotein, along with a second variant that contained the nanoLuciferase (nLuc) gene in place of accessory gene 7a (Fig. 1A), using a D614-form SARS-CoV-2 strain WA1 as the backbone (*8*). To examine whether the D614G substitution enhances authentic SARS-CoV-2 entry, we infected four susceptible cell lines with the ancestral wild-type (WT)–nLuc and D614G-nLuc viruses and maintained in the medium containing neutralizing antibodies (Abs) to limit viral spreading. Luciferase signals representing initial entry events were measured at 8 hours after infection (Fig. 1B). In accord with pseudovirus studies (*4*, *9*), the D614G-nLuc infection resulted in a 3.7- to 8.2-fold higher transgene expression as compared with WT-nLuc virus in different cell lines. Growth curves comparing WT and D614G viruses were performed in those cell lines (Fig. 1C). Although the D614G variant showed similar or slightly higher titers at the early time point (8 hours), its peak titers were ~0.5 log units lower than the ancestral WT virus in Vero-E6 and A549-ACE2 cell lines but not in Vero-81 and Huh7.

To evaluate the replication of SARS-CoV-2 D614G variant in the human respiratory tract, we compared the multistep growth kinetics [multiplicity of infection (MOI) = 0.1] of the WT and D614G viruses in ex vivo primary human nasal epithelial (HNE) cells from five donors, large (proximal) airway epithelial (LAE) cells from four donors, and distal lung small airway epithelial (SAE) cells from three donors. Cultures from the same donor were infected with either WT or D614G virus in triplicate (Fig. 1, D to F, and fig. S1, A and B). Both viruses infect mainly ciliated cells in the primary pulmonary cultures (fig. S1C). Paired *t*-test analysis suggests that the D614G-infected HNE cells at 24, 48, and 72 hours and LAE cultures at 48 hours exhibited statistically higher titers than those infected with the WT virus. This enhanced replication was not observed at any time points in distal lung SAE cultures derived from three donors. To further compare replication fitness between the two variants, competition assays were performed in LAE cultures by infecting simultaneously with both viruses (Fig. 1G). After three continuous passages at 72-hour intervals, the D614G variant became dominant in the cultures regardless of whether the WT virus was at a 1:1 or 10:1 ratio over the isogenic D614G mutant (Fig. 1, H and I). Taken together, these data suggest the D614G substitution enhances SARS-CoV-2 replication fitness in the primary epithelial cells, with an advantage in the upper respiratory tract epithelial cells in nasal and large (proximal) airway epithelia that express higher amounts of human ACE2 (hACE2) receptor (*8*).

**Fig. 2:**
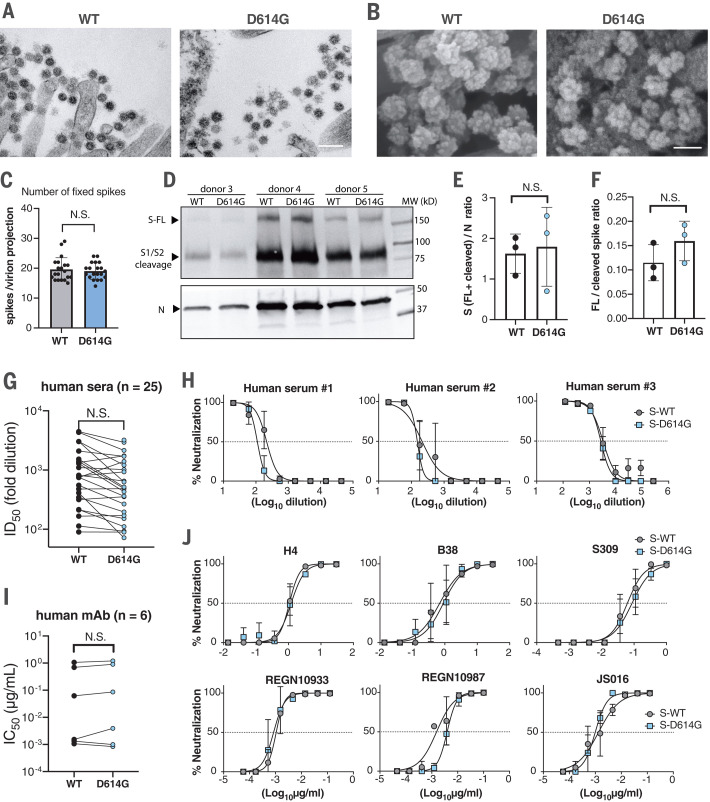
D614G substitution does not alter SARS-CoV-2 virion morphology, S protein cleavage patterns, and sensitivity to neutralizing Abs. (**A**) TEM image of WT and D614G virions on airway epithelial cell surface. Scale bar, 200 nm. (**B**) SEM images of WT and D614G virions on airway epithelial cell surface. Scale bar, 100 nm. (**C**) Quantification of S protein on individual SARS-CoV-2 virion projections. The number of S proteins on individual virion projections from different SEM images was quantified manually, *n* = 20. (**D**) Western blot analysis of SARS-CoV-2 virions washed from WT- or D614G-infected HNE cultures at 72 hours after infection. Each lane contains a pooled sample from triplicated cultures derived from the same donor. Full-length (FL), S1/S2 cleaved spike protein (S), and nucleocapsid protein (N) were probed. Samples in each pair were loaded on the basis of an equal amount of the N protein. MW, molecular weight. (**E** and **F**) S-to-N ratios (E) and FL-to-cleaved S ratios (F) were determined by measuring relative intensity of bands in the Western blot image. (**G**) Summary of ID_50_ values of 25 convalescent human sera against WT- and D614G-nLuc viruses. (**H**) Neutralization curves of three representative human sera. Viral sequence reveals that serum #1 was collected from a COVID-19 patient infected with a D614G SARS-CoV-2 variant. (**I** and **J**) Summarized IC_50_ values (I) and individual neutralization curves (J) of six human neutralizing mAbs against both viruses. Data in (C), (E), and (F) are indicated as mean ± SD and were analyzed by unpaired *t* test; data in (G) and (I) were analyzed by paired *t* test. N.S., not significantly different.

Next, scanning and transmission electron microscopy (SEM and TEM) were performed to visualize virions present on the surface of primary human airway cell cultures. No significant differences in virion morphology were detected (Fig. 2, A and B). The number of spike proteins on individual virion projections was not significantly different between the two viruses in the EM images (Fig. 2C). Western blot analysis also shows similar spike-to-nucleocapsid ratios between the two viruses in samples collected from multiple HNE cultures (Fig. 2, D and E). Differences in spike cleavage were also not observed between the two viruses (Fig. 2, D and F). Further, we evaluated the neutralization properties of convalescent human serum samples (*n* = 25) using the nLuc-expressing recombinant SARS-CoV-2 encoding either WT or D614G spike (Fig. 2, G and H). The samples show similar half-maximal inhibitory dilution (ID_50_) values against both viruses. Similarly, six RBD-binding, SARS-CoV-2–neutralizing monoclonal antibodies (mAbs) showed no significant difference at half-maximal inhibitory concentration (IC_50_) values against both viruses (Fig. 2, I and J). Together, these data suggest that the D614G substitution does not significantly alter SARS-CoV-2 morphology, spike cleavage pattern, and in vitro neutralization properties in the context of live virus.

**Fig. 3:**
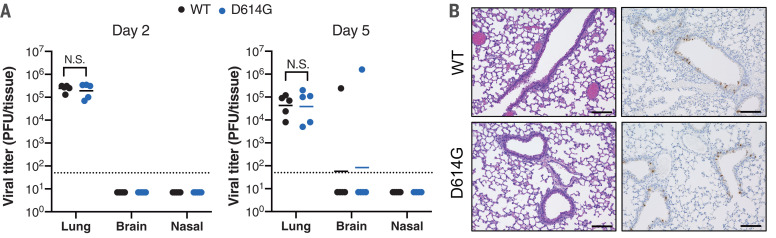
D614G substitution does not alter SARS-CoV-2 pathogenesis in hACE2 mice. (**A**) Lung, brain, and nasal turbinate titers of WT and D614G-infected mice were determined on day 2 and day 5. Each mouse was infected with 10^5^ PFU of the virus (*n* = 5 per group); the plaque assay detection limit (1.7 log_10_PFU/ml) is indicated as dashed lines. Data were analyzed by unpaired *t* test. (**B**) Representative hematoxylin and eosin (H&E) staining and IHC staining of SARS-CoV-2 N protein in the lung tissues collected from infected hACE2 mice harvested at day 2 after infection. Scale bars, 100 μm.

**Fig. 4:**
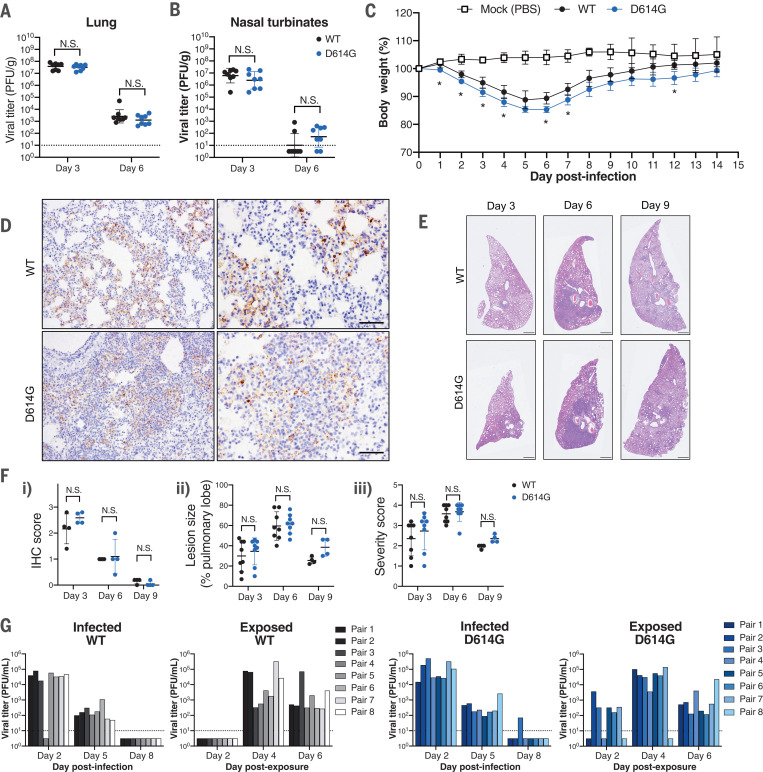
**D614G substitution enhances SARS-CoV-2 transmission in hamsters.** (**A** and **B**) Viral titers in the lung (A) and nasal turbinates (B) collected from SARS-CoV-2–infected hamsters at days 3 and 6. Each hamster was infected with 10^3^ PFU of the virus, *n* = 8 per virus for each time point; the plaque assay detection limit (1 log_10_PFU/g) is indicated as dashed lines. (**C**) Body weight of mock-, WT-, and D614G-infected hamsters (*n* = 4 per group). Hamsters in the body weight study were not subjected to nasal wash sampling. *p < 0.05 (**D**) IHC staining of SARS-CoV-2 nucleocapsid proteins in representative lung tissues collected from WT- and D614G-infected hamsters at day 3. Scale bars, 100 μm. (**E**) H&E staining of representative lung tissues collected on days 3, 6, and 9 from hamsters infected with WT or D614G. Scale bars, 1 mm. (**F**)****(i) Quantification of IHC-positive cells in hamster lung tissues, using the following scoring system: 0, no positive cell; 1, <10%; 2, 10 to 50%; and 3, >50% positive cells in each lobe of lung. (ii) The size of pulmonary lesions was determined on the basis of the mean percentage of affected area in each section of lobes from each animal. (iii) Pathological severity scores in infected hamsters, based on the percentage of inflammation area for each section of the five lobes collected from each animal using the following scoring system: 0, no pathological change; 1, affected area (≤10%); 2, affected area (<50% and >10%); and 3, affected area (≥50%). An additional point was added when pulmonary edema and/or alveolar hemorrhage was observed. (**G**) Viral titers in nasal washes collected from infected and exposed hamster pairs in WT and D614G groups; plaque assay detection limit (1 log_10_PFU/ml) is indicated as dashed lines. The number of positive hamsters in both exposure groups at day 2 (WT versus D614G = 0 of 8 versus 5 of 8) was analyzed by Fisher’s exact test, *P* = 0.0256. N.S., not significantly different.

To evaluate the role of the D614G substitution in viral pathogenesis, we infected *hACE2* transgenic mice and Syrian hamsters with equal plaque-forming units (PFU) of WT or D614G viruses. SARS-CoV-2 infection in *hACE2* mice exhibited a mild disease phenotype, characterized by high viral titers in lung and brain tissues but minimum weight loss and undetectable nasal titers (*10*). Two groups of *hACE2* mice infected with WT or D614G viruses exhibited undetectable viral titers in nasal turbinates and similar lung viral titers at days 2 and 5 after infection. One mouse (out of 5) from both groups exhibited detectable viral titers in the brain (Fig. 3A). Histopathological analyses revealed similar numbers of lesions and SARS-CoV-2–infected cells in the mouse lung tissue harvested at day 2 after infection (Fig. 3B). With respect to hamster studies, lung and nasal turbinate tissues collected at days 3 and 6 after infection exhibited similar viral titers in each group (Fig. 4, A and B). However, the D614G-infected hamsters lost slightly more body weight than those infected with the WT virus (Fig. 4C). Immunohistochemistry (IHC) showed similar amounts of viral antigen staining in the hamster lung tissue collected at days 3, 6, and 9 from both groups (Fig. 4, D and Fi). Histopathological examination revealed similar severe pulmonary lesions with inflammatory cell infiltration in the alveolar walls and air spaces, pulmonary edema, and alveolar hemorrhage in both of the hamsters on day 3, extended across larger areas on day 6, and then exhibiting partial resolution by day 9 (Fig. 4E). There was no significant difference in the size of the lung lesions (Fig. 4Fii) and the histological severity (Fig. 4Fiii). To evaluate the roles of the D614G variant replication fitness in vivo, we performed a competition assay in four independent lines of hamsters. Each hamster was infected with 1000 PFU of a mixture containing a 1:1 ratio of both viruses (fig. S2B). After three continuous passages in naïve animals at 3-day intervals, we observed that the D614G variant became dominant in the lung tissues of animals after the first passage of all groups (fig. S2, C and D), which is consistent with the phenotype of enhanced fitness of the D614G virus noted in the human LAE competition assay. These studies indicate that the D614G substitution contributes to marginal enhancement of SARS-CoV-2 pathogenesis in hamsters, but not in *hACE2* mice, and to improved competitive fitness in the hamster model.

To evaluate the impact of the D614G substitution in SARS-CoV-2 respiratory transmissibility, we set up eight pairs of hamsters for each virus similar to previous studies (*11*, *12*). Each pair comprised a naïve hamster adjacent to a cage with an infected animal 1 day after infection (fig. S2, E and F). Viral titers in the nasal wash samples from all animals were monitored. Both WT and D614G viruses were transmitted efficiently to naïve hamsters, as evidenced by positive nasal wash samples detected in all exposed animals at day 4 (Fig. 4G). The infected groups at all three time points and the exposure groups at days 4 and 6 exhibited similar viral titers between the two viruses. However, five of eight hamsters exposed to the D614G-infected group showed infection and detectable viral shedding at day 2, whereas those exposed to the WT-infected group had no infection and viral shedding (*P* = 0.0256, Fisher’s exact test), supporting the hypothesis that the D614G variant transmits significantly faster than the WT virus between hamsters.

Emerging viruses, such as CoVs, alphaviruses, and filoviruses, have undergone sequential rounds of evolution while adapting to the new human hosts in epidemic or pandemic settings (*13*–*15*). Among CoVs, mutations in the spike glycoprotein have been associated with altered pathogenesis, receptor usage, and neutralization (*16*–*19*), potentially challenging the development of vaccine and therapeutic Abs that are urgently needed at present. The emergent D614G mutation in the spike gene of SARS-CoV-2 strains has raised concerns about potential enhancements in transmissibility, antigenicity, and/or pathogenesis. Using authentic SARS-CoV-2 isogenic variants, we show the role of the D614G substitution in enhancing viral infectivity in immortalized cell lines, growth, and fitness in primary human airway epithelial cells and hamsters, yet it marginally alters viral pathogenesis in hamster and hACE2 mouse models. We demonstrate that the D614G variant transmits significantly faster between hamsters through aerosol and droplets.

Recent studies indicate that D614G alters spike trimer hydrogen-bond interactions, reorienting the RBD into an “up” conformation and increasing ACE2 receptor binding and infectivity (*7*, *20*). Consistent with previous pseudotype virus studies (*4*, *9*, *21*, *22*), our data show that the D614G recombinant virus enters immortalized cell lines more efficiently than the WT virus. However, we did not observe the enhancement of viral titers in continuous replication kinetics, suggesting that the variable ACE2 and protease concentrations between different cell lines and the virion thermostability may also affect the D614G replication in vitro. The efficient replication and fitness in our ex vivo models suggest that SARS-CoV-2 D614G isogenic virus displays a notable advantage in epithelial cells in the nose and upper respiratory tract. These data support the role of the nasal epithelium and the D614G substitution in enhanced infectivity and transmission in human populations (*3*).

Patients infected with the D614G virus have not been conclusively linked to increased disease severity (*3*, *4*). In this study, we evaluated the pathogenesis of the D614G variants in both *hACE2* mouse and hamster models. Equivalent virus titers were measured in the lungs and nasal turbinates of all time points, and a similar severity of lesions was observed in the histopathological samples, suggesting that the D614G substitution does not significantly enhance the SARS-CoV-2 pathogenesis in both animal models, although this phenotype needs to be confirmed in both sexes of animals in future studies. However, the increased weight loss and improved in vivo replication fitness in hamsters suggest that the D614G variant may cause marginally enhanced disease outcomes. Although complicated by the presence of other mutations in the spike, these differences may become more evident in a lethal SARS-CoV-2 infection model in young, adult, or aged mice in future studies (*23*). In the hamster transmission study, the D614G isogenic transmitted significantly faster to adjacent animals early in infection, showing that the substitution preserved efficient transmission in vivo. As SARS-CoV-2 replicates preferentially in the nasal and olfactory epithelium, depending on differences in ACE2 and TMPRSS2 cell type expression patterns across species (*8*, *24*, *25*), these data are consistent with a model of increased replication in the nasal epithelium and large airway epithelium, leading to enhanced virus growth compared with the ancestral virus and more efficient transmissibility. Potential reasons for this phenotype could be that the D614G variant exhibits a lower minimum infectious dose to animals and/or to subtle variations in virion stability in small and large droplets, which requires further mechanistic studies in the future.

Using pseudotype viruses, the D614G substitution has been suggested to increase proteolytic cleavage and S glycoprotein incorporation into virions, reduce S1 loss, and promote enhanced infectivity in vitro (*4*, *9*, *21*). Against the backdrop of a full complement of SARS-CoV-2 structural proteins, our study demonstrated no obvious differences in proteolytic processing or S incorporation into isogenic virions encoding the D614G mutations, perhaps reflecting differences in S trimer incorporation and presentation between authentic and pseudotyped viruses; the latter lack a full component of virion proteins. The effect of the D614G variant on vaccine efficacy has been of major concern. Consistent with previous studies (*5*, *22*), we showed overall equivalent sensitivity of the both luciferase reporter viruses to the 25 convalescent human sera and 6 RBD-binding mAbs, suggesting that the D614G substitution does not significantly shift SARS-CoV-2 neutralization properties. Some sera and mAbs, such as serum #1 and REGN10987, displayed slightly different neutralization potencies against the two viruses, suggesting subtle differences in the Ab binding properties. As a limitation, the virus genotype in most serum donors remains unknown. These data also suggest that the current vaccine approaches directed against WT spike should be effective against the D614G strains. The relationship between increased transmission and virulence remains complex and could be affected by age, sex, and other comorbidities, and it is unclear whether the minimum infectious dose may be lower for D614G in humans (*26*). It is important to monitor and identify the emergence of new variants of SARS-CoV-2 with increased transmission and pathogenesis and/or altered antigenicity, especially as levels of human herd immunity and active interventions alter the selective forces that operate on the genome.

## Supplementary Materials

science.sciencemag.org/content/370/6523/1464/suppl/DC1 Materials and Methods Figs. S1 and S2 References (*27*–*30*) MDAR Reproducibility Checklist

## Appendix

View/request a protocol for this paper from *Bio-protocol*.
